# OD45 Evaluating Quality In Health Economics: Quality Appraisal Checklist For Systematic Reviews Of Studies Eliciting Health State Utility Values

**DOI:** 10.1017/S0266462324001727

**Published:** 2025-01-07

**Authors:** Muchandifunga T. Muchadeyi, Karla Hernandez-Villafuerte, Gian Luca Di Tanna, Rachel Eckford, Yan Feng, Michela Meregaglia, Tessa Peasgood, Stavros Petrou, Jasper Ubels, Michael Schlander

## Abstract

**Introduction:**

The reliability of cost–utility analyses depends on the quality of health state utility values (HSUVs). Given the increasing number of studies eliciting HSUVs, systematic reviews (SRs) are vital to economic evaluations. Nevertheless, a universally acceptable quality appraisal (QA) tool specific to the SRs of HSUV studies is lacking—this study aimed to develop one and fill this gap.

**Methods:**

We employed a mixed-method approach, starting with a rapid review to identify QA dimensions, QA items, and terminology in the SRs of HSUV-eliciting studies. This informed a modified Delphi process with a seven-member international expert panel, aiming to define key terms, refine the QA tool dimensions, and establish relevant signaling questions. The experts participated in two anonymous online survey rounds interspersed with structured feedback, enabling iterative refinement of their views. Following these surveys, a virtual face-to-face meeting was held to resolve outstanding issues. Consensus was defined a priori at all stages of the modified Delphi process.

**Results:**

The rapid review identified three QA dimensions and 16 initial items, noting the diverse terminologies in defining QA. Response rates to the first- and second-round questionnaires and the virtual consensus meeting were 100, 86, and 71 percent, leading to a consensus on the definitions of scientific quality, QA, the three QA dimensions (reporting, methodological limitation, and risk of bias and relevance), and scope of the QA tool. The number of QA items was refined to 14: all relevant to reporting, six to relevance, and 11 to methodological limitations and bias risk dimensions. The QA tool underscores distinct evaluations for each dimension.

**Conclusions:**

We present the first version of a QA checklist designed to provide SR authors with a tool to appraise the quality of HSUV-eliciting studies comprehensively. The QA tool aims to (i) facilitate QA in SRs of HSUV elicitation studies, (ii) promote consistency in the appraisal process, and (iii) emphasize the importance of differentiating between reporting quality, methodology, and relevance.

